# Preliminary assessment of pre‐morbid DNA methylation in individuals at high genetic risk of mood disorders

**DOI:** 10.1111/bdi.12415

**Published:** 2016-07-21

**Authors:** Rosie May Walker, Jessika Elizabeth Sussmann, Heather Clare Whalley, Niamh Margaret Ryan, David John Porteous, Andrew Mark McIntosh, Kathryn Louise Evans

**Affiliations:** ^1^ Medical Genetics SectionCentre for Genomic and Experimental MedicineInstitute of Genetics and Molecular MedicineThe University of EdinburghWestern General HospitalEdinburghUK; ^2^ Division of PsychiatryThe University of EdinburghRoyal Edinburgh HospitalUniversity of EdinburghEdinburghUK; ^3^ Centre for Cognitive Ageing and Cognitive EpidemiologyThe University of EdinburghEdinburghUK

**Keywords:** 450K array, bipolar disorder, DNA methylation, *Interleukin 1 Receptor Accessory Protein‐Like 1*, major depressive disorder, premorbid, *Transcription Factor 4*

## Abstract

**Objectives:**

Accumulating evidence implicates altered DNA methylation in psychiatric disorders, including bipolar disorder (BD) and major depressive disorder (MDD). It is not clear, however, whether these changes are causative or result from illness progression or treatment. To disentangle these possibilities we profiled genome‐wide DNA methylation in well, unrelated individuals at high familial risk of mood disorder. DNA methylation was compared between individuals who subsequently developed BD or MDD [ill later (IL)] and those who remained well [well later (WL)].

**Methods:**

DNA methylation profiles were obtained from whole‐blood samples from 22 IL and 23 WL individuals using the Infinium HumanMethylation450 BeadChip. Differential methylation was assessed on a single‐locus and regional basis. Pathway analysis was performed to assess enrichment for particular biological processes amongst nominally significantly differentially methylated loci.

**Results:**

Although no locus withstood correction for multiple testing, uncorrected *P*‐values provided suggestive evidence for altered methylation at sites within genes previously implicated in neuropsychiatric conditions, such as *Transcription Factor 4* (*TCF4*) and *Interleukin 1 Receptor Accessory Protein‐Like 1* ([*IL1RAPL1*]; *P≤*3.11×10^−5^). Pathway analysis revealed significant enrichment for several neurologically relevant pathways and functions, including *Nervous System Development and Function* and *Behavior*; these findings withstood multiple testing correction (*q*≤0.05). Analysis of differentially methylated regions identified several within the major histocompatibility complex (*P*≤.000 479), a region previously implicated in schizophrenia and BD.

**Conclusions:**

Our data provide provisional evidence for the involvement of altered whole‐blood DNA methylation in neurologically relevant genes in the aetiology of mood disorders. These findings are convergent with the findings of genome‐wide association studies.

Mood disorders, comprising bipolar disorder (BD) and major depressive disorder (MDD), represent a leading cause of disability, causing considerable suffering and decreased productivity. Both conditions are heritable^1^, ^2^ and genetic risk factors for the two conditions overlap.^3^ First‐degree relatives of individuals diagnosed with BD are at an increased risk of developing both BD (relative risk=5.8–7.9) and MDD (relative risk=2.1).^1^ Whilst there is a clear genetic component to BD and MDD, environmental risk factors also play an important role, as revealed by imperfect monozygotic twin concordance.^4^, ^5^, ^6^

DNA methylation, the addition of a methyl group to the fifth carbon atom of cytosine, normally in the context of a CpG dinucleotide, can play a role in transcriptional regulation.^7^ Altered gene expression has been widely reported in patients with mood disorders^8^, ^9^, ^10^ and recent studies support the existence of DNA methylation changes in individuals with these conditions,^11^, ^12^, ^13^, ^14^, ^15^ implying a potential mechanistic link. Importantly, DNA methylation is under the control of both genetic and environmental factors,^16^, ^17^ rendering it a plausible candidate mechanism for mediating the deleterious effects of experiences such as obstetric complications, antenatal maternal virus and childhood neglect, which are known to increase risk for mood disorders.^18^, ^19^, ^20^ Consistent with this possibility, differences in DNA methylation have been observed in monozygotic twins who are discordant for BD^11^ or MDD.^12^, ^13^

The evidence for altered DNA methylation in individuals with mood disorders comes from the study of individuals who are ill and, usually, medicated. It is, therefore, impossible to determine whether observed methylation differences play a primary role in development of mood disorders or reflect the effects of illness progression and/or treatment. The likely confounding effect of medication has been demonstrated by recent studies showing the modulation of DNA methylation by antidepressants and mood stabilizers.^21^, ^22^, ^23^ Another important limitation when considering the evidence for altered DNA methylation in mood disorders is that the majority of studies have profiled DNA from peripheral tissues, such as blood. This reflects the practical difficulties of obtaining DNA from the more aetiologically relevant brain. Within‐individual correlation between blood and brain methylation levels has been shown to be limited for the majority of sites, although there are some exceptions.^24^ This observation does not, however, detract from the potential utility of blood‐based methylation signatures as a biomarker for mood disorders. Moreover, it is possible that, when considered at a pathway level, differences in DNA methylation in the blood might indicate the types of biological processes perturbed in mood disorders.

Here, we sought to circumvent some of these potential confounds by profiling whole‐blood genome‐wide DNA methylation in the unaffected first‐ or second‐degree relatives of individuals with BD, who are at increased risk of developing mood disorders themselves (henceforth referred to as high‐risk individuals). These individuals were recruited as part of the Edinburgh‐based Scottish Bipolar Family Study (BFS). Studies of this cohort have identified a number of differences in brain structure, function and connectivity in individuals at high risk of developing mood disorders.^25^, ^26^ Moreover, in a recent functional magnetic resonance imaging study, increased insula activation during a sentence completion task was found to be predictive of future illness in well high‐risk individuals.^27^ In addition, we have recently shown altered miRNA expression in high‐risk individuals,^28^ thus highlighting altered regulation of gene expression as a putative risk mechanism. Blood samples for DNA methylation profiling were obtained upon recruitment to the cohort and the diagnostic status of participants evaluated biennially, permitting baseline DNA methylation to be compared between unaffected individuals who later developed a mood disorder (ill later [IL]) and those who remained well (well later [WL]).

## 1 Patients and materials

### 1.1 Sample

Genome‐wide DNA methylation was profiled in whole‐blood samples obtained from 49 individuals at higher genetic risk of developing a mood disorder, all of whom were unaffected at the time of blood draw. These individuals were recruited as part of the Scottish Bipolar Family Study (BFS), as described previously.^25^, ^26^ Briefly, at the time of recruitment, high‐risk individuals met the following selection criteria: (i) at least one first‐degree or two second‐degree relatives with bipolar I disorder, (ii) no personal history of BD, and (iii) aged 16–25 y. Exclusion criteria were: a history of major depression, mania, hypomania, psychosis, generalized anxiety disorder, panic disorder, eating disorder, or substance dependence; an IQ<70 or clinical diagnosis of learning disability; any major neurological disorder or history of head injury that included loss of consciousness; and any contraindications to magnetic resonance imaging (MRI). Only unrelated individuals were included. All participants provided written informed consent and the study was approved by the Multicentre Research Ethics Committee for Scotland and performed in accordance with the Helsinki Declaration of 1975.

### 1.2 Clinical assessments

Baseline clinical assessments were carried out at the time of each participant's blood draw. All individuals were well at the time of recruitment. Follow‐up assessments have been performed at approximately 2‐y intervals since the baseline assessment. Clinical interviews were conducted by two psychiatrists. At baseline, current depressive and manic symptoms were assessed using the Hamilton Depression Rating Scale (HDRS)^29^ and the Young Mania Rating Scale (YMRS),^30^ respectively. At follow‐up interviews, participants were reassessed using the Structured Interview for DSM‐IV to determine whether they had developed a mood disorder (MDD or BD). When participants were unable to attend follow‐up assessments, diagnosis was made on the basis of information regarding current clinical care, obtained through contact with the National Health Service (*n*=8; of whom two were diagnosed with a mood disorder and six remained well). On the basis of their diagnosis at follow‐up assessments, the high‐risk individuals were split into two groups: a group who remained well at every subsequent follow‐up assessment (WL) and a group who had developed a mood disorder (IL). Statistical differences in HDRS and YMRS scores were assessed by Kruskal‐Wallis tests. Statistical differences in the length of follow‐up time (defined as the number of years between the baseline assessment and either [i] the assessment where a participant was diagnosed with either MDD or BD for the IL group or [ii] the most recent follow‐up assessment for the WL group) were assessed by one‐tailed *t* test to determine whether the IL group had been followed up for longer than the WL group. Statistical significance was defined as *P*≤.05.

### 1.3 Extraction of blood DNA

Blood (9 mL) was collected in an EDTA tube. DNA was extracted at the Wellcome Trust Clinical Research Facility (WTCRF) at the University of Edinburgh, using the Nucleon BACC2 Genomic DNA Extraction Kit (Fisher Scientific, Loughborough, UK), following the manufacturer's instructions.

### 1.4 Genome‐wide methylation profiling

Whole‐blood genomic DNA (500 ng) was treated with sodium bisulphite using the EZ‐96 DNA Methylation Kit (Zymo Research, Irvine, CA), following the manufacturer's instructions. DNA methylation was assessed using the Infinium HumanMethylation450 BeadChip (Illumina Inc., San Diego, CA), according to the manufacturer's protocol. Samples were assigned to chips such that, as far as possible, group and gender were counterbalanced across chips.

Raw intensity (.idat) files were read into R using the *minfi* package,^31^ which was used to perform initial quality control assessments. Subsequently, filtering of poor‐performing samples and sites was performed. Samples were removed from the dataset if: (i) they failed any of the quality control assessments carried out in *minfi*; or (ii) ≥1% sites had a detection *P*‐value of >.05. Probes were removed from the dataset if: (i) the CpG site (and/or the base before the CpG site, for Type I probes) overlapped a single nucleotide polymorphism (SNP) with a minor allele frequency of ≥5% in the European population from the 1000 Genomes Project^32^, ^33^; (ii) they were predicted to cross‐hybridize^33^; (iii) they had more than five samples with a beadcount of <3; or (iv) ≥0.5% samples had a detection *P*‐value of >.05.

The data were normalized using the dasen method from the R package *wateRmelon*.^34^ This involves adjusting the background difference between Type I and Type II assays (by adding the offset between Type I and II probe intensities to Type I intensities). Between‐array quantile normalization is then performed for the methylated and unmethylated signal intensities separately (Type I and Type II assays normalized separately).

Prior to downstream analyses, M‐values, defined as M=log2([M+100]/[U+100]), where M represents the methylated signal intensity and U represents the unmethylated signal intensity, were calculated for the normalized data.

### 1.5 Assessment of between‐group differences in whole‐blood cellular composition

In order to assess between‐group differences in cellular composition, estimated cell counts for B‐lymphocytes, granulocytes, monocytes, natural killer cells, CD4+ T‐lymphocytes and CD8+ T‐lymphocytes were generated using the estimateCellCounts() function in *minfi*. This function implements Jaffe and Irizarry's^35^ modified version of Houseman's^36^ algorithm. Between‐group differences in cell composition were assessed using a Student's *t* test. A *P‐*value of ≤.05 was deemed to represent a significant between‐group difference.

### 1.6 Surrogate variable analysis

DNA methylation can be influenced by many sources of variation, making it important to account for these variables when assessing differential methylation. A complicating factor is that many sources of variation are unknown or unmeasured. Even when potential sources of variation are measured, it is not always clear how best to model these potential confounding variables.^37^ Surrogate variable analysis (SVA) identifies a set of significant surrogate variables (SVs), the linear combination of which represents variation in DNA methylation that is not attributable to the primary variable(s) of interest. When fitted as covariates in the linear models implemented to identify differentially methylated positions (DMPs), the SVs control for sources of unmeasured/unmodelled variation (e.g. cell composition and smoking status), which might otherwise confound the relationship between DNA methylation and the independent variable of interest.^37^, ^38^ An advantage of SVA is that it allows for complex relationships between confounders and DNA methylation, for example interactions between confounding variables. SVA was carried out using the *“be”* method with the R package SVA,^39^ fitting group (IL or WL) as the primary variable of interest and age and gender as adjustment variables. It should be noted that no distinction was made between IL individuals who later developed MDD and those who developed BD when estimating surrogate variables.

### 1.7 Identification of differentially methylated positions

DMPs were identified using the R package *limma* 40 by fitting linear models with the outcome variable “M‐value” and the predictor variables “group” (IL or WL), “gender,” and “age” together with the significant SVs. Correction for multiple testing was implemented using the Benjamini‐Hochberg false discovery rate (FDR), with *q*‐values of ≤0.05 deemed to be significant.

### 1.8 Assessment of the correlation between blood and brain methylation

For loci of interest, an indicator of the likely correlation between blood and brain methylation was obtained using the recently developed Blood Brain DNA Methylation Comparison Tool (http://epigenetics.iop.kcl.ac.uk/bloodbrain/ 24). This tool permits the user to investigate the correlation between DNA methylation in the blood and in four different brain regions (entorhinal cortex [ERC], prefrontal cortex [PFC], superior temporal gyrus [STG] and cerebellum [CBM]), using matched samples obtained from 71–75 individuals who were assessed using the Infinium HumanMethylation450 BeadChip. Pearson's correlation coefficients obtaining a *P*‐value ≤.05 were considered statistically significant.

### 1.9 Pathway analysis

Ingenuity Pathway Analysis (IPA) (Ingenuity Systems, Redwood City, CA; www.ingenuity.com) was used to identify canonical pathways (defined as manually curated, well‐characterized metabolic and cell signalling pathways), diseases and biological functions that were enriched amongst the genes containing methylation sites that attained an uncorrected *P*‐value of ≤.01 (*n*=3272). IPA assesses enrichment using the right‐tailed Fisher's exact test. As multiple hypotheses were tested, the Benjamini‐Hochberg FDR method was implemented to calculate *q*‐values. We defined statistical significance as *q*≤0.05. The list of genes targeted by probes that were included in the DMP analysis was used as the reference set (*n*=22 255).

### 1.10 Identification of differentially methylated regions

Differentially methylated regions (DMRs) were identified using a modified version of the champ.lasso function implemented in the R package *ChAMP*.^41^, ^42^ DMRs were defined as regions containing three or more adjacent probes within a defined lasso region showing unidirectional changes in methylation that attained nominal significance (*P*≤.05) in the DMP analysis. The lasso region was set to 2 kb and was scaled according to the local genomic/epigenomic landscape.^41^ According to champ.lasso's default settings, DMRs were merged with neighbouring DMRs where they were separated by less than 1 kb. *P*‐values were estimated for each DMR according to the method described by Butcher et al.^41^. Briefly, individual probe *P*‐values were combined using Stouffer's method, weighting each probe *P*‐value by the underlying correlation structure of the M‐values. *P*‐values from correlated probes were down‐weighted whilst *P*‐values from uncorrelated probes were up‐weighted. A DMR *P*‐value meeting a Benjamini‐Hochberg FDR‐corrected threshold of ≤.05 was required for a DMR to be included in the final DMR lists.

## 2 Results

### 2.1 Quality control and data filtering

In order to mitigate potential confounds, 7219 probes affected by SNPs and 30 165 probes predicted to cross‐hybridize^33^ were removed from the dataset. The overall success of DNA methylation profiling was then assessed using the internal quality control probes. This revealed incomplete bisulphite conversion in two samples, which were omitted from downstream analyses. Finally, 11 182 probes were removed as they had either (i) more than five samples with a beadcount <3 or (ii) ≥0.5% samples with a detection *P*‐value of >.05. Two samples were excluded as >1% sites had a detection *P*‐value >.05. At the end of this filtering process, the dataset comprised 437 239 probes measured in 23 individuals who remained well (WL) and 22 individuals who later developed a mood disorder (IL).

### 2.2 Sample demographics

Genome‐wide DNA methylation was profiled using the Infinium HumanMethylation450 BeadChip in whole‐blood samples from individuals at high familial risk of developing a mood disorder who were well at the time of blood sampling. Of the 45 well high‐risk individuals successfully profiled in this study, 22 have subsequently developed either MDD or BD (MDD=19; BD=3) and 23 have remained well. These two groups are referred to as “ill later” (IL) and “well later” (WL), respectively. Sample demographic information is presented in Table 1, with further diagnostic information available in Table S1. There were no significant differences between the groups in terms of gender, age, cigarette smoking, or depressive or manic symptoms. Importantly, the IL group had not been followed up for longer than the WL group (mean follow‐up years: WL=4.67, IL=2.68, *t*=−4.41, *P*>.99).

**Table 1:** Sample demographic information

Variables	WL (*n*=23)	IL (*n*=22)	Test statistic	*P*‐value
Gender, % male	56.5	45.5	NA^e^	.556
Age, y^a^	21.7 (2.77)	21.4 (3.22)	*t*=−.344	.732
HDRS score^b^	0 (0–1.75)	0 (0–9)	*W*=115	.176
YMRS score^b^	0 (0–0)	0 (0–0)	*W*=99	.502
Smoker, % yes^c^^,^ d	27.3	50.0	NA^e^	.215
If yes, cigarettes/day^b^	11 (4.75–14)	6 (2–15)	*W*=22.5	.313
Follow‐up time, y^a^^,^ f	4.67 (1.76)	2.68 (1.18)	*t*=−4.41	>.990

HDRS, Hamilton Depression Rating Scale; IL, ill later (well high‐risk individuals who developed a mood disorder); NA, not applicable; WL, well later (well high‐risk individuals who remained well); YMRS, Young Mania Rating Scale.

a Group means and standard deviations for normally distributed variables.

b Group medians and interquartile ranges for non‐normally distributed variables.

c Percentage “yes” for categorical variables.

d Smoking data were not available for one WL participant.

e Fisher's exact test.

f One‐tailed *t* test.

### 2.3 Assessment of between‐group differences in cell composition

As whole blood is composed of multiple different cell types, each characterized by a specific DNA methylation profile, variation in the cellular composition of blood samples can confound epigenome‐wide association studies.^35^ We therefore carried out a between‐group comparison of the estimated proportions of six blood cell subtypes. A significant between‐group difference was identified in one cell type, CD4+ T‐cell proportions (*t*=2.87; *P*=.006 35; for complete results see Table S2). In the presence of low levels of cellular heterogeneity, SVA can be used to empirically control for cell type composition.^35^

### 2.4 Surrogate variable analysis

In order to control for the potentially confounding effects of unmeasured or unmodelled variables, SVs were estimated using the R package SVA.^39^ SVA identified 11 significant SVs. The linear combination of these SVs represents the combined effects of any confounding variables that exert a systematic effect on DNA methylation, such as variation in cellular composition.

### 2.5 Assessment of differential methylation

Differential methylation was assessed at 437 239 sites that survived quality control filtering. The presence of DMPs was assessed by linear models that included the predictor variables group (IL or WL), age, gender and 11 significant surrogate variables. Inspection of a quantile‐quantile plot of observed vs expected *P*‐values did not indicate any systematic bias in the data (Fig. S1), consistent with the genomic inflation factor of 1.01. The 20 most significantly differentially methylated loci identified in the IL group compared to the WL group are shown in Table 2 (see Table S3 for the top 100 loci; see Fig. S2 for plots of the top 10 loci). No locus remained significant after correction for multiple testing; however, it was of interest that loci mapping to genes previously implicated in neuropsychiatric conditions were amongst those loci with the most significant unadjusted *P*‐values. These genes included *Transcription Factor 4* (*TCF4*) (*P*=3.11×10^−5^), variation in which has been associated with schizophrenia in genome‐wide association analysis,^43^ and *Interleukin 1 Receptor Accessory Protein‐Like 1* (*IL1RAPL1*) (*P*=1.77×10^−5^), disruption/deletion of which has been identified in individuals with mental retardation and/or autism spectrum disorder (ASD).^44^, ^45^, ^46^, ^47^, ^48^, ^49^, ^50^ No relationship was found between methylation and estimated CD4+ T‐cell proportion at either the *TCF4* site or the *IL1RAPL1* site (*TCF4*:* t*=−1.22, *P*=.230; *IL1RAPL1*:* t*=1.38, *P*=.176).

**Table 2:** The top 20 differentially methylated positions identified in the well high‐risk individuals who developed a mood disorder (ill later [IL]) compared to the well high‐risk individuals who remained well (well later [WL]), ranked by uncorrected *P*‐value

Probe ID	Gene symbol	Chr.	Coordinate^a^	*P*‐value	WL β (mean)	IL β (mean)	β difference^b^
cg07398767	*CA10*	17	50237205	3.72×10^−6^	0.384	0.332	−0.053
cg08292919	*GABBR1*	6	29565696	4.22×10^−6^	0.900	0.880	−0.020
cg18995182	*COL4A5; COL4A6*	X	107682860	4.62×10^−6^	0.386	0.350	−0.036
cg19006127	—	4	3794737	5.97×10^−6^	0.205	0.187	−0.018
cg08608800	*ZC3H13*	13	46626937	6.91×10^−6^	0.159	0.138	−0.021
cg19199483	*—*	13	111483035	1.26×10^−5^	0.960	0.954	−0.007
cg01642827	*GET4*	7	925664	1.30×10^−5^	0.919	0.910	−0.009
cg03773183	—	17	79325994	1.33×10^−5^	0.781	0.854	0.074
cg01724150	*NMNAT3*	3	139397363	1.64×10^−5^	0.413	0.347	−0.066
cg26893134	*FRK*	6	116381904	1.73×10^−5^	0.914	0.893	−0.021
cg06927864	*IL1RAPL1*	X	28604661	1.77×10^−5^	0.278	0.343	0.065
cg19298821	*C5orf48*	5	125967357	2.00×10^−5^	0.918	0.932	0.013
cg00662273	*PDPK1*	16	2648122	2.03×10^−5^	0.932	0.921	−0.011
cg10827045	*PPT1*	1	40562842	2.09×10^−5^	0.092	0.101	0.009
cg06352873	*DCBLD2*	3	98596709	2.33×10^−5^	0.841	0.821	−0.020
cg08883995	*MGMT*	10	131389152	2.37×10^−5^	0.911	0.923	0.012
cg24507551	*GTF2H4*	6	30877958	2.67×10^−5^	0.896	0.908	0.011
cg08187750	*PIWIL1*	12	130824529	2.68×10^−5^	0.674	0.620	−0.055
cg02328223	*MGLL*	3	127541927	3.08×10^−5^	0.097	0.110	0.013
cg15171911	*TCF4*	18	53068921	3.11×10^−5^	0.713	0.671	−0.042

a GRCh37/hg19.

b IL β mean–WL β mean.

CA10: Carbonic Anhydrase 10; GABBR1: Gamma‐Aminobutyric Acid Type B Receptor Subunit 1; COL4A5: Collagen Type IV Alpha 5; COL4A6: Collagen Type IV Alpha 6; ZC3H13: Zinc finger CCCH domain‐containing protein 13; GET4: Golgi To ER Traffic Protein 4; NMNAT3: Nicotinamide Nucleotide Adenylyltransferase 3; FRK: Fyn Related Src Family Tyrosine Kinase; IL1RAPL1: Interleukin 1 Receptor Accessory Protein Like 1; C5orf48: Chromosome 5 Open Reading Frame 48; PDPK1: 3‐Phosphoinositide Dependent Protein Kinase 1; PPT1: Palmitoyl‐Protein Thioesterase 1; DCBLD2: Discoidin, CUB And LCCL Domain Containing 2; MGMT: O‐6‐Methylguanine‐DNA Methyltransferase; GTF2H4: General Transcription Factor IIH Subunit 4; PIWIL1: Piwi Like RNA‐Mediated Gene Silencing 1; MGLL: Monoglyceride Lipase; TCF4: Transcription Factor 4.

As DNA methylation was assessed in the blood rather than in the brain, it was important to establish whether the nominally significant differences in methylation we observed in the blood might indicate the presence of methylation differences in the brain. By assessing DNA methylation in matched blood and brain samples from 71–75 individuals, Hannon et al.^24^ have generated an online database (http://epigenetics.iop.kcl.ac.uk/bloodbrain/) of coefficients for the correlation between DNA methylation in the blood and four different brain regions (PFC, ERC, STG, and CBM). Blood−brain correlation coefficients for each of the 20 most significantly differentially methylated loci are presented in Table S4. Ten of these loci were found to show a significant correlation between methylation in the blood and methylation in at least one brain region. Using the criteria defined by Hannon et al.,^24^ whole‐blood methylation at one of these ten loci showed strong correlation (explaining at least 50% of the variance) and methylation levels at an additional three loci showed moderate correlation (explaining at least 20% of the variance) with methylation in at least one brain region.

Of particular note, methylation at the locus mapping to *IL1RAPL1* is significantly correlated between the blood and all four brain regions (*P*‐value and correlation range: *P*=1.02×10^−8^; *r* ^2^=.381 [ERC]–*P*=5.7×10^−6^; *r* ^2^=.259 [CBM]). Three other loci (mapping to *Carbonic Anhydrase‐Related Protein 10* [*CA10*], *Collagen Type IV*,* Alpha 5/6* [*COL4A5*/*COL4A6*], and an intergenic region on chromosome 17) show significant correlation between the blood and all four brain regions, with the correlations involving the loci mapping to *COL4A5*/*COL4A6* and the intergenic region showing moderate and strong correlations with brain methylation, respectively. An additional locus, mapping to *Piwi‐Like RNA‐Mediated Gene Silencing 1* (*PIWIL1*), is significantly correlated between the blood and the three cortical regions, explaining a moderate proportion of the variance in methylation in each brain region.

In order to ascertain whether genes involved in particular biological pathways and functions were over‐represented amongst those harbouring loci showing the most significant differences in methylation, pathway analysis was performed. This resulted in the identification of several pathways and functions pertinent to nervous system function and current hypotheses regarding the pathogenesis of mood disorders (Table 3). These included the canonical pathways “Dopamine‐DARPP32 Feedback in cAMP Signalling” (*q*=2.31×10^−2^) and “Wnt/β‐catenin Signalling” (*q*=3.07×10^−2^), and the functions “Nervous System Development and Function” (best subcategory *q*=3.71×10^−11^) and “Behaviour” (best subcategory *q*=4.69×10^−5^). This analysis was carried out using a *P*‐value threshold of *P*≤.01 to define the target list. For comparison, and to assess the robustness of these findings, pathway analysis was carried out using two additional *P*‐value thresholds, *P*≤.005 and *P*≤.001 (Tables S5 and S6). “Behaviour” was significantly enriched in both additional analyses and “Nervous System Development and Function” was significantly enriched when using a threshold of *P*≤.005.

**Table 3:** Summary of pathway analysis results

Name	*P*‐value	*q*‐value	Ratio^a^
Canonical pathways			
Dopamine‐DARPP32 (Dopamine‐ and cAMP‐regulated phosphoprotein, Mr 32 kDa) feedback in cAMP signalling	4.33×10^−5^	2.31×10^−2^	47/155 (0.303)
PI3K signalling in B lymphocytes	1.21×10^−4^	3.07×10^−2^	38/122 (0.311)
Wnt/β‐catenin signalling	2.28×10^−4^	3.07×10^−2^	47/165 (0.285)
Endometrial cancer signalling	2.30×10^−4^	3.07×10^−2^	20/52 (0.385)
Protein kinase A signalling	4.18×10^−4^	3.27×10^−2^	89/368 (0.242)

Name	*P*‐value range	*q*‐value range	No. molecules
Molecular and cellular functions			
Cell‐to‐cell signalling and interaction	1.72×10^−15^–5.15×10^−3^	3.71×10^−11^–2.61×10^−1^	225
Cellular assembly and organization	1.72×10^−15^–6.83×10^−3^	3.71×10^−11^–3.01×10^−1^	520
Cell death and survival	8.83×10^−8^–6.27×10^−3^	1.00×10^−4^–2.87×10^−1^	924
Cell morphology	2.67×10^−6^–6.57×10^−3^	1.20×10^−3^–2.97×10^−1^	662
Cellular development	8.43×10^−6^–7.42×10^−3^	3.30×10^−3^–3.19×10^−1^	898
Physiological system development and function			
Nervous system development and function	1.72×10^−15^–7.42×10^−3^	3.71×10^−11^–3.19×10^−1^	620
Tissue morphology	1.72×10^−15^–7.24×10^−3^	3.71×10^−11^–3.15×10^−1^	582
Behaviour	3.48×10^−8^–6.64×10^−3^	4.69×10^−5^–2.97×10^−1^	283
Embryonic development	5.97×10^−7^–7.24×10^−3^	4.37×10^−4^–3.15×10^−1^	515
Organismal development	5.97×10^−7^–6.64×10^−3^	4.37×10^−4^–2.97×10^−1^	711
Diseases and disorders			
Cancer	6.66×10^−11^–6.57×10^−3^	2.06×10^−7^–2.97×10^−1^	2714
Gastrointestinal disease	6.66×10^−11^–4.88×10^−3^	2.06×10^−7^–2.61×10^−1^	2042
Organismal injury and abnormalities	6.66×10^−11^–7.26×10^−3^	2.06×10^−7^–3.15×10^−1^	2717
Hepatic system disease	5.72×10^−9–^2.01×10^−8^	1.24×10^−5^–2.89×10^−5^	1185
Hereditary disorder	5.79×10^−7^–6.88×10^−3^	4.37×10^−4^–3.01×10^−1^	187

a The ratio indicates the number of molecules associated with a particular pathway, disease or function in the target set compared to the reference set.

### 2.6 Identification of differentially methylated regions

To identify regions of the genome harbouring multiple (≥3) nominally significant DMPs (*P*≤.05), DMR analysis was performed. This resulted in the identification of 61 DMRs (Table 4). Interestingly, seven DMRs are located within the major histocompatibility complex (MHC; *P*‐value range: 1.81×10^−7^–4.79×10^−4^), a region that has been implicated in the pathogenesis of schizophrenia and BD.^43^, ^51^ Amongst the other DMRs identified, a region of hypermethylation encompassing an intronic and exonic region of *Msh Homeobox 1* (*MSX1*;* P*=1.81×10^−6^) is noteworthy in light of previous evidence for altered methylation of this gene in BD and schizophrenia.^52^

**Table 4:** Differentially methylated regions (DMRs) identified in the well high‐risk individuals who developed a mood disorder (IL) compared to the well high‐risk individuals who remained well (WL), ranked by *P*‐value

Gene symbol	*P*‐value	Chr.	Start coordinate^a^	DMR size (bp)	No. probes	Direction^b^
*S100A13*;* S100A1*	4.90×10^−9^	1	153599240	1024	8	Up
*SIAH3*	1.22×10^−8^	13	46425082	994	6	Up
*PIWIL2*	2.80×10^−8^	8	22132877	176	6	Down
*MIR146B*	3.53×10^−8^	10	104195449	2184	5	Down
*NA*	1.26×10^−7^	10	50648574	2766	5	Down
*NA*	1.81×10^−7^	6	30094974	458	8	Up
*FRK*	2.19×10^−7^	6	116381824	177	4	Down
*FOXR1*	9.96×10^−7^	11	118842395	98	3	Down
*DSN1*	1.27×10^−6^	20	35402286	23	3	Up
*MSX1*	1.81×10^−6^	4	4864284	292	4	Up
*STRA6*	1.92×10^−6^	15	74494622	526	3	Up
*FMN1*	1.92×10^−6^	15	33360245	216	3	Down
*IGF2BP1*	1.92×10^−6^	17	47092126	292	3	Down
*CRISP2*	3.13×10^−6^	6	49680994	478	5	Down
*ZFYVE28*	5.75×10^−6^	4	2402943	2179	4	Up
*NA*	8.56×10^−6^	10	96989451	3146	4	Down
*C10orf82*	1.02×10^−5^	10	118429409	86	3	Up
*LOC400794*;* LRRC52*	1.29×10^−5^	1	165512892	895	4	Up
*TCEAL8*	1.61×10^−5^	X	102510031	169	3	Down
*NTNG1*	1.61×10^−5^	1	108023288	378	4	Down
*NA*	1.84×10^−5^	14	75799632	2483	3	Down
*NA*	1.88×10^−5^	6	164505176	3163	4	Down
*SNX29*	2.29×10^−5^	16	12183512	1047	3	Down
*NA*	2.29×10^−5^	1	2885051	200	4	Up
*NA*	3.05×10^−5^	8	2584143	3174	4	Down
*APC2*	3.53×10^−5^	19	1466930	180	3	Up
*NA*	3.53×10^−5^	17	76640024	2564	3	Down
*NA*	3.62×10^−5^	20	60540386	166	3	Down
*RASA3*	3.73×10^−5^	13	114829829	910	3	Down
*NA*	4.35×10^−5^	11	85392601	2386	3	Down
*NA*	4.55×10^−5^	6	32862234	199	3	Down
*GABRG3*	4.95×10^−5^	15	27215925	73	3	Down
*EIF3I*;* C1orf91*	6.10×10^−5^	1	32687540	77	3	Down
*RPP21*	7.36×10^−5^	6	30312957	166	3	Up
*NA*	8.01×10^−5^	10	54537213	2561	3	Down
*NA*	8.06×10^−5^	6	33866229	3244	4	Up
*C6orf27*	8.06×10^−5^	6	31733373	1607	5	Up
*NA*	0.000 101	16	22959736	222	3	Down
*PARP9*;* DTX3L*	0.000 101	3	122283571	128	4	Up
*IQSEC1*	0.000 123	3	13082461	852	3	Up
*DDR1*	0.000 138	6	30854046	302	3	Up
*NA*	0.000 139	7	75702611	2518	3	Down
*UBE2U*	0.000 148	1	64669387	70	3	Down
*MDH2*;* STYXL1*	0.000 160	7	75677907	398	3	Down
*NA*	0.000 169	5	134880228	3484	3	Up
*PRRT1*	0.000 198	6	32117135	388	3	Up
*NA*	0.000 198	13	21900230	324	3	Up
*NA*	0.000 214	3	195681893	3484	3	Down
*CMTM2*	0.000 231	16	66613264	18	3	Up
*MCTS1*	0.000 256	X	119737646	562	3	Down
*STC2*	0.000 261	5	172749612	2495	3	Down
*NA*	0.000 275	7	27137009	3484	3	Up
*NA*	0.000 368	16	1157350	3484	3	Down
*GJB6*	0.000 441	13	20805265	241	3	Up
*NA*	0.000 441	12	120835638	166	3	Up
*AIF1*	0.000 479	6	31583077	1984	3	Up
*TNFAIP2*	0.000 519	14	103593425	173	3	Down
*LSM5*;* AVL9*	0.000 711	7	32535138	78	3	Down
*NA*	0.000 803	6	33129826	324	3	Down
*TNFRSF11A*	0.000 946	18	60052280	368	3	Up
*NA*	0.000 978	16	86252489	2708	3	Up

a GRCh37/hg19.

b “Up” indicates hypermethylation in the IL group; “Down” indicates hypomethylation in the IL group.

S100A13: S100 calcium‐binding protein A13; S100A1: S100 calcium‐binding protein A1; SIAH3: Siah E3 Ubiquitin Protein Ligase Family Member 3; PIWIL2: Piwi Like RNA‐Mediated Gene Silencing 1; MIR146B: miRNA 146B; FRK: Fyn Related Src Family Tyrosine Kinase; FOXR1: Forkhead Box R1; DSN1: DSN1 Homolog, MIS12 Kinetochore Complex Component; MSX1: Msh Homeobox 1; STRA6: Stimulated By Retinoic Acid 6; FMN1: Formin 1; IGF2BP1: Insulin Like Growth Factor 2 MRNA Binding Protein 1; CRISP2; Cysteine‐Rich Secretory Protein 2; ZFYVE28: Zinc Finger FYVE‐Type Containing 28; C10orf82: Chromosome 10 Open Reading Frame 82; LOC400794: uncharacterized LOC400794; LRRC52: Leucine Rich Repeat Containing 52; TCEAL8: Transcription Elongation Factor A (SII)‐Like 8; NTNG1: Netrin G1; SNX29: Sorting Nexin 29; APC2: Adenomatosis Polyposis Coli 2; RASA3: RAS P21 Protein Activator 3; GABRG3: gamma‐aminobutyric acid (GABA) A receptor, gamma 3; EIF3I: Eukaryotic Translation Initiation Factor 3 Subunit I; C1orf91: Chromosome 1 Open Reading Frame 91; RPP21: Ribonuclease P/MRP 21kDa Subunit; C6orf27: Chromosome 6 Open Reading Frame 27; PARP9: Poly(ADP‐Ribose) Polymerase Family Member 9; DTX3L: Deltex 3 Like, E3 Ubiquitin Ligase; IQSEC1: IQ Motif And Sec7 Domain 1; DDR1: Discoidin domain receptor family, member 1; UBE2U: Ubiquitin Conjugating Enzyme E2 U (Putative); MDH2: Malate Dehydrogenase 2; STYXL1: Serine/Threonine/Tyrosine Interacting‐Like 1; PRRT1: Proline‐Rich Transmembrane Protein 1; CMTM2: CKLF‐Like MARVEL Transmembrane Domain Containing 2; MCTS1: Malignant T‐Cell Amplified Sequence 1; STC2: Stanniocalcin 2; GJB6: Gap junction beta‐6 protein; AIF1: Allograft inflammatory factor 1; TNFAIP2: TNF Alpha Induced Protein 2; LSM5: LSM5 Homolog, U6 Small Nuclear RNA And MRNA Degradation Associated; AVL9: AVL9 Cell Migration Associated; TNFRSF11A: Tumor Necrosis Factor Receptor Superfamily Member 11a.

The correlation between methylation in the blood and in four different regions for probes contributing to the 10 most significant DMRs was investigated using Hannon et al.'s^24^ online database. These coefficients are presented in Table S7. All 10 DMRs contained at least one probe that showed a significant correlation with at least one brain region. Of these ten DMRs, evidence for moderate/strong correlation for at least one probe in at least one brain region was seen for six DMRs. For two DMRs, significant correlation was observed between methylation in the blood and all four brain regions for all probes contributing to the DMR. These two DMRs are located (i) in the first exon/intron of one *Piwi‐Like RNA‐Mediated Gene Silencing 2* (*PIWIL2*) isoform and in the promoter region of another *PIWIL2* isoform (*P*‐value and correlation range: 2.32×10^−9^; *r* ^2^=.406–.000 355; *r* ^2^=.163) and (ii) in the promoter region of *DSN1, MIS12 Kinetochore Complex Component* (*DSN1*;* P*‐value and correlation range: 3.64×10^−14^; *r* ^2^=.622–.000 661; *r* ^2^=.156).

## 3 Discussion

Accumulating evidence suggests that changes in DNA methylation might play a role in psychiatric illnesses, including the mood disorders MDD and BD.^11^, ^12^, ^13^, ^14^, ^15^, ^53^ Due to the fact that studies carried out to date have involved participants who are already ill and usually medicated, it has not been possible to determine whether altered DNA methylation occurs before illness onset or as a consequence of illness progression or in response to treatment.

To attempt to disentangle these possibilities, we characterized blood‐based DNA methylation in a cohort of well individuals at elevated familial risk of developing a mood disorder who were separated by future diagnostic status into a group that remained well and a group that became ill. Comparison of these two groups did not identify any methylation differences at individual loci that survived FDR correction; however, uncorrected *P*‐values provided suggestive evidence for altered DNA methylation at a number of sites throughout the genome. Pathway analysis of these sites revealed FDR‐significant enrichment for altered methylation at sites within genes involved in several neurologically relevant pathways believed to contribute to the pathogenesis of mood disorders.

The fact that none of the site‐specific differences in DNA methylation survived correction for multiple testing likely reflects both the limited statistical power conferred by the study of a small sample and issues surrounding the application of multiple testing corrections to epigenome‐wide association studies (EWASs). The small size of our sample reflects the difficulties inherent in assessing premorbid differences and means that our findings must be considered exploratory and in need of replication. Regarding the second issue, multiple testing correction of EWASs is complicated by a lack of variability in methylation at many CpGs and the existence of spatial correlation between the methylation levels of nearby sites.^16^ These factors are likely to render many multiple testing correction procedures overly conservative.^54^ In light of these limitations, we took the decision to present and discuss uncorrected *P*‐values for the single‐site differential methylation analysis. As such, it is important to consider the possibility that our failure to identify any FDR‐significant differences reflects a true lack of such differences and it is imperative that this caveat is borne in mind when considering the loci of interest discussed below.

Amongst the loci showing the most significant changes in DNA methylation, several sites map to genes previously implicated in psychiatric illness and/or with a known neurological function. One example is a site within the promoter region of *IL1RAPL1* where we observed a nominally significant increase in methylation in IL individuals. *IL1RAPL1* encodes the X‐linked interleukin‐1 receptor accessory protein‐like 1, which is highly expressed in the brain, particularly in the hippocampus,^48^ and has been found to mediate the formation of excitatory synapses.^55^ *Il1rapl1* knock‐out mice show reduced cortical neuron spine density, mild memory impairments, increased locomotor activity and reduced anxiety‐like behaviours.^56^ Deletions and point mutations in *IL1RAPL1* have been identified in individuals with mental retardation and individuals with ASD with and without mental retardation.^44^, ^45^, ^46^, ^47^, ^48^, ^49^, ^50^

Importantly, methylation at this locus has previously been shown to be significantly correlated between the blood and four different brain regions.^24^ The differentially methylated site is located within a DNAse hypersensitivity site (DHS),^57^ indicating the presence of an open chromatin structure. As such, it is possible that the altered methylation observed at this site might exert an effect on *IL1RAPL1* expression.

Another nominally significant DMP rendered particularly interesting by significant blood−brain correlation falls within the first intron of *PIWIL1*. Methylation at this site is significantly correlated between the blood and three cortical regions.^24^ PIWIL1 is best known for its role in maintaining germline integrity by repressing retrotransposon activity^58^; however, a recent study has shown that it also plays a role in the polarization and migration of neurons in the developing cerebral cortex.^59^ This site is located within a DHS and a chromatin immunoprecipitation (ChIP)‐identified binding site for the transcription factor TCF7L2,^57^ suggesting a potential regulatory effect.

We identified hypomethylation in IL individuals at a site located within *TCF4*. This site falls within a region of *TCF4* implicated in the pathogenesis of schizophrenia by a recent genome‐wide association study (GWAS).^43^ Moreover, a cross‐disorder GWAS, which assessed association in individuals with schizophrenia, BD, MDD, autism spectrum disorders or attention deficit‐hyperactivity disorder, found suggestive evidence for association with a variant in *TCF4*, which fell just short of genome‐wide significance.^60^ *TCF4* encodes a basic helix‐loop‐helix transcription factor, which plays a role in multiple processes, including neurodevelopment.^61^

In order to investigate the potential biological implications of the observed methylation changes, we carried out pathway analysis. In addition to aiding in the interpretation of our results, this approach confers the advantage of mitigating the effects of individual false positive results, a feature that is particularly important in light of the failure of the single‐locus results to withstand correction for multiple testing. Many of the significantly enriched biological pathways and functions that withstood correction for multiple testing pertained to neurologically relevant functions. These categories included *Nervous System Development and Function* and *Behavior*.

A limitation of pathway analysis of methylation data is that a single *P*‐value must be selected to represent each gene. IPA, by default, selects the most significant *P*‐value for any probe within a given gene. Key to this rationale is the fact that it is possible for altered methylation at one locus to impact on gene function. As such, genes that contain more CpG sites are more likely to obtain a more significant *P*‐value because it is more likely that their function will be altered by a change in methylation. Genes containing more CpG sites are also more likely, however, to obtain a significant *P*‐value by chance due to multiple testing. The inter‐relationship between these two factors presents an analytical challenge, as correcting for multiple testing risks reducing true biological signal. It is not clear how best to simultaneously account for both factors using currently available pathway analysis methods, meaning this limitation must be taken into account when interpreting our findings.

In addition to considering our data at the level of individual loci, we carried out DMR analysis to identify regions containing multiple, adjacent differentially methylated loci. Several of the DMRs identified mapped to the MHC (*n*=7) or the broader extended MHC (an additional two DMRs). The most significant association in the Psychiatric Genomics Consortium's latest GWAS of schizophrenia^43^ was within the MHC and a GWAS involving a combined schizophrenia and BD case group also identified association with this region.^51^ These genetic findings are in keeping with evidence from clinical observations, and animal and cellular models that implicate perturbed immune function in the pathogenesis of mood disorders.^62^, ^63^

Among the other DMRs identified, a 272‐bp region of hypermethylation spanning an intronic and exonic region at the 5__ end of *MSX1* is of particular interest. In a recent study of DNA methylation at sites within γ‐aminobutyric acid‐related genes in the post‐mortem hippocampus of individuals with schizophrenia or BD, the majority of significant DMPs and DMRs were found to map to *MSX1*.^52^ *MSX1* encodes a transcription factor that is involved in regulating central nervous system development, including the differentiation of midbrain dopaminergic neurons.^64^, ^65^ Hannon et al.^24^ identified a significant positive correlation between methylation in the blood and the STG for three of the four probes that form this DMR.

A region of hypomethylation found to affect the first exon/intron of one *PIWIL2* isoform and the promoter region of another *PIWIL2* isoform was of particular interest given the consistently significant positive correlation between methylation in blood and all four brain regions observed by Hannon et al.^24^ for the five loci involved in this DMR. Taken together with our finding of hypomethylation at the *PIWIL1* locus, this result suggests that further investigation of the role of the PIWI pathway in neuronal function and psychiatric illness may be warranted.

In common with pathway analysis, a key advantage to DMR analysis is that it serves to reduce the influence of individual false positive results; however, there are some important limitations to DMR analysis that must be considered. The ability to detect DMRs is affected by the representation of a given genomic region on the Infinium HumanMethylation450 BeadChip. Although the method we utilized to detect DMRs does attempt to account for this, it is clear that DMPs that are located in regions that are otherwise probe deserts will never be detected through DMR analysis. A pertinent example is the *TCF4* probe cg15171911: although this probe showed nominally significant differential methylation in our single‐locus analysis, it could not contribute to a DMR as the nearest site represented in our dataset is located ~79 kb away. As such, the results of DMP and DMR analyses should be considered complementary. A second issue surrounding the detection of DMRs is that decisions must be made regarding several parameters, such as the number of adjacent probes required to form a DMR, the significance level that must be attained by individual probes contributing to the DMR, and the width of the window in which to search for DMRs. At this time, there is no standard definition of a DMR, limiting the ability to compare DMR results between studies.

Our analysis concerns a broad diagnostic category, termed *mood disorder*, which comprises both MDD and BD. The majority of individuals in the group who later developed a mood disorder were diagnosed with MDD; however, it is likely that some of these individuals will later develop BD. A longitudinal study of the offspring of parents with BD has previously shown that the majority of those who eventually developed BD received a first diagnosis of MDD.^66^ The mean period between the onset of MDD and the appearance of the first (hypo)manic episode was 4.9 y. Follow‐up of the individuals involved in the BFS is ongoing, thus permitting future studies to address questions relating to more specific diagnostic categories, once the diagnostic statuses of the participants have stabilized.

We profiled DNA methylation in whole blood, a heterogeneous tissue comprising multiple cell types. As each blood‐cell subtype is characterized by a distinct methylation profile, it is possible for variation in blood composition to confound studies of whole‐blood DNA methylation.^36^, ^67^ As cell count data were not available for our samples, we estimated the proportions of six blood cell subtypes and compared these proportions between groups. We identified a significant difference in the proportion of CD4+ T cells. Since estimates of blood cell proportions are made with an associated error, it is unclear how they should be used to control for differences in blood cell composition. Recently, Jaffe and Irizarry (2014) recommended using SVA to control for cell‐type composition effects when levels of confounding are low. We therefore utilized SVA to minimize confounding by cell composition variation. Additionally, for two DMPs of particular interest, *TCF4* and *IL1RAPL1*, we demonstrated that there was no relationship between methylation level and the estimated proportion of CD4+ T cells.

In addition to accounting for the effects of variation in cellular composition on DNA methylation, SVA also controls for any other unmeasured/unmodelled variables that exert a systematic effect on DNA methylation. As such, we have controlled for the confounding effects of variables such as cigarette smoking, which alter methylation.^68^ Since SVA operates in a hypothesis‐free manner, it negates the need to decide on the most appropriate measure of a variable to fit as a covariate. This is particularly advantageous when considering a complex behaviour, such as smoking, which can be described in many ways, including multiple aspects of current smoking behaviour, former smoking behaviour and exposure to the smoke of others.

A perennial issue in the study of psychiatric disorders is the difficulty in accessing the most physiologically relevant tissue (i.e. the brain). This prompts a key and unresolved question regarding the utility of studying DNA methylation in a peripheral tissue, such as the blood. Clearly, the importance of the blood−brain correlation depends on whether peripheral methylation differences are being assessed for their potential to act as a biomarker of illness (i.e. a *signature* of illness) or as a key to understanding the underlying pathophysiology of the condition (i.e. a *mirror* of methylation differences in the brain).^69^ Clearly, covariation between blood and brain methylation is only necessary in the latter case. A recent study has found within‐individual correlation between DNA methylation in the blood and the brain to be low for the majority of sites measured by the Infinium HumanMethylation450 BeadChip; however, there are some exceptions.^24^ Examination of blood−brain correlation coefficients^24^ for the loci of interest identified here suggests the possibility of a mixture of “signature” and “mirror” effects. The enrichment for several neurologically relevant terms in our pathway analysis results suggests, however, that, despite extensive between‐tissue variation, when considered together methylation differences measured in the blood might be capable of highlighting potential pathogenic mechanisms.

## 4 Conclusions

Here we report the first genome‐wide characterization of DNA methylation in individuals prior to the onset of mood disorders and, through the identification of affected genes and pathways, elucidate potential biological mechanisms through which altered methylation might confer risk. Importantly, the IL individuals did not score higher than the WL group on measures of depressive or manic symptoms at the time that their blood sample was obtained. This indicates that the DNA methylation changes observed do not simply reflect the presence of an undiagnosed mood disorder at the time of the blood draw. As we compared two groups of individuals who were both at an elevated familial risk of developing a mood disorder, the changes in DNA methylation we observed may reflect premorbid dysregulation in the group who later became ill or protective changes in the group who remained well. Future studies profiling DNA methylation at a second time point may aid in disentangling these mechanisms. The current study was under‐powered to address the question of whether premorbid DNA methylation differs between individuals who develop MDD and those who develop BD; however, this would be an interesting question to address in the future. Our findings add to a recent imaging study involving an overlapping set of individuals from the BFS cohort, which identified increased activity in the insula cortex, a brain region involved in emotional processing, during a sentence completion task in the individuals who developed MDD.^27^ Together, these studies highlight the value of longitudinal cohort studies for addressing questions of causality, which typically plague case–control studies.

## Disclosures

HCW has received research funding from Pfizer. DJP has received research funding from Eli Lilly & Co. and Janssen. AMM has received research funding from Pfizer, Eli Lilly & Co. Janssen, and SACCADE Diagnostics. KLE has received research funding from Eli Lilly & Co. and Janssen. None of this funding contributed to work carried out in the present study. RMW, JES, and NMR do not have any potential conflicts of interest to report.
