# Supplementary material for: Preliminary assessment of pre‐morbid DNA methylation in individuals at high genetic risk of mood disorders
Source: Bipolar Disord. 2016 Jul 21;18(5):410–22. doi: 10.1111/bdi.12415 (PMC5006843; doi:10.1111/bdi.12415)
Supplement: Supplementary file 1 [file BDI-18-410-s001.docx]

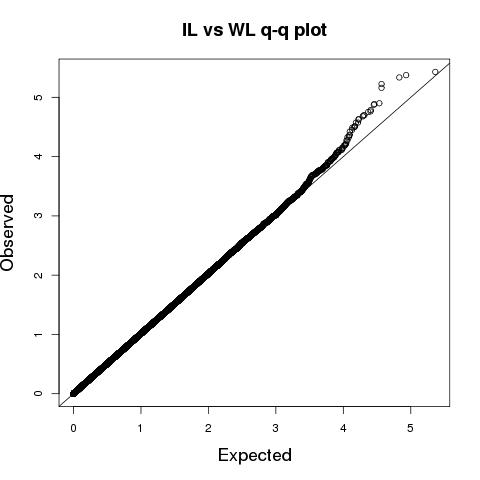


λ = 1.01

**Supplementary figure 1.** Quantile-quantile plot. The x-axis shows –log_10_ transformed observed p-values and the y-axis indicates –log_10_ transformed expected *p*-values. The lambda inflation factor (median[observed]/median[expected]) is shown.
