# Supplementary material for: Preliminary assessment of pre‐morbid DNA methylation in individuals at high genetic risk of mood disorders
Source: Bipolar Disord. 2016 Jul 21;18(5):410–22. doi: 10.1111/bdi.12415 (PMC5006843; doi:10.1111/bdi.12415)
Supplement: Supplementary file 2 [file BDI-18-410-s002.pdf]

cg07398767 (*CA10*)

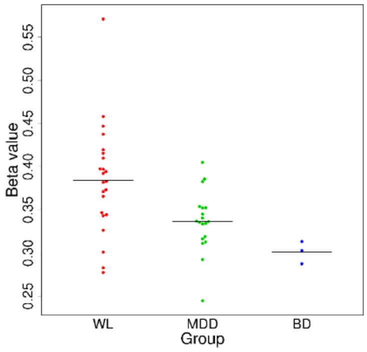

cg08292919 (*GABBR1*)

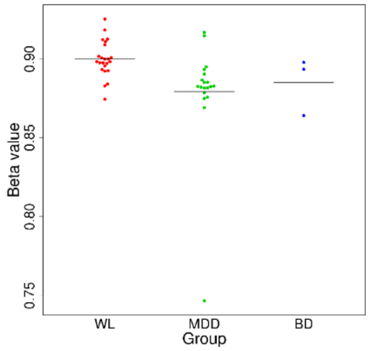

cg18995182 (*COL4A5/6*)

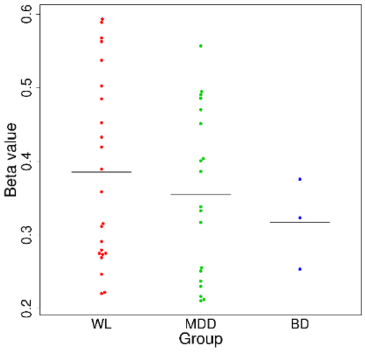

cg19006127 (*N/A*)

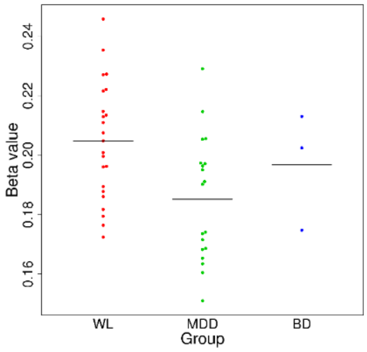

cg08608800 (*ZC3H13*)

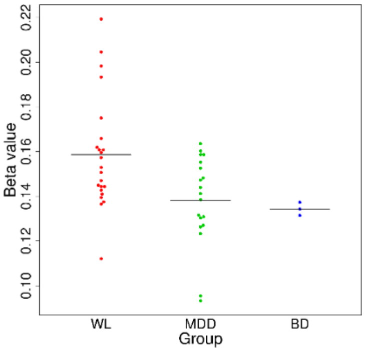

cg19199483 (*N/A*)

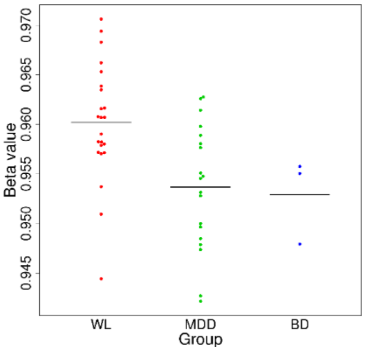

cg01642827 (*GET4*)

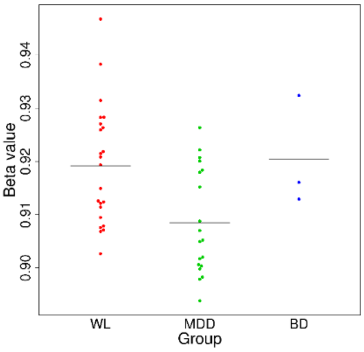

cg03773183 (*N/A*)

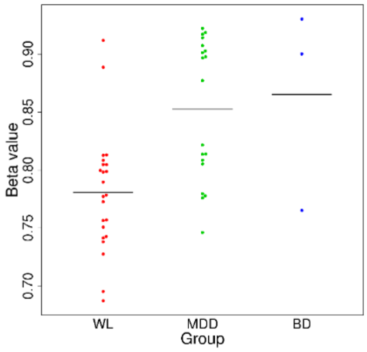

cg01724150 (*NMNAT3*)

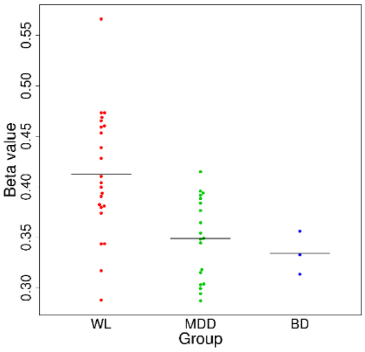

cg26893134 (*FRK*)

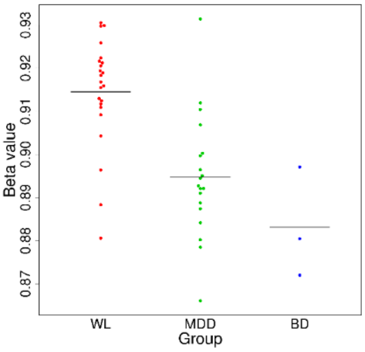

Supplmentary Figure 2. Beeswarm plots showing methylation beta-values for the top ten differentially methylated loci (ranked by uncorrected *p*-value) identified when comparing high-risk individuals who remained well (WL) to those who developed either bipolar disorder (BD) or major depressive disorder (MDD). For illustration purposes, beta-values are plotted separately according to future diagnosis; all analyses were, however, carried out using a combined “ill later (MDD and BD)” group.
